# Case for diagnosis. A transient unilateral face rash upon eating: Frey syndrome^☆^

**DOI:** 10.1016/j.abd.2021.10.013

**Published:** 2022-11-02

**Authors:** Ana Gusmão Palmeiro, Laura Azurara, Bernardo Pimentel, Cristina Amaro

**Affiliations:** a Department of Dermatology, Centro Hospitalar Lisboa Ocidental, Hospital de Egas Moniz, Lisboa, Portugal; b Department of Pediatrics, Centro Hospitalar Lisboa Ocidental, Hospital de São Francisco Xavier, Lisboa, Portugal

Dear Editor,

A 9-months-old infant, born at full term and delivered with the assistance of forceps, presented to the Dermatology clinic with a history of flushing and warming on his right cheek that appeared when he ate (Fig. 1). The flushing first become apparent to his parents at 6-months of age, upon introduction of solid foods. It usually began within seconds of ingesting the first bite and resolved in 30 minutes. It was not associated with hyperhidrosis, pruritus, discomfort, lip swelling, wheezing, or dyspnea. The patient had not undergone surgery, nor had he had a personal history of atopy.

**Figure 1:**
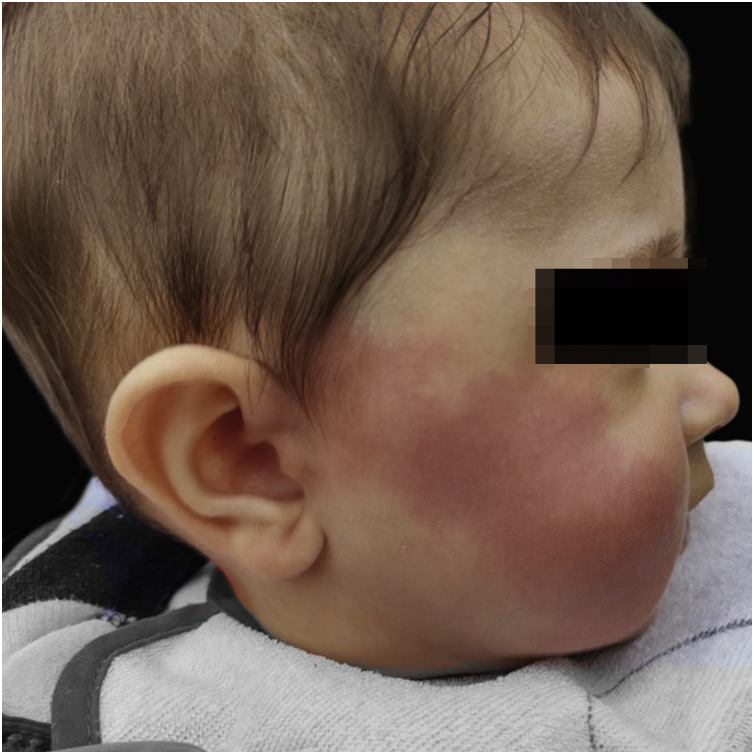
Flushing of the right cheek.

At the initial observation in the clinic, there were no skin lesions, but within seconds after a provocation test by eating a strawberry, unilateral erythema and localized warmth appeared across his right zygomatic arch region, malar eminence, and submalar region (Fig. 2). The remainder dermatologic exam was normal and there were no associated signs or symptoms, including sweating. The rash faded after around 15 minutes. The neurological exam was unremarkable.

**Figure 2:**
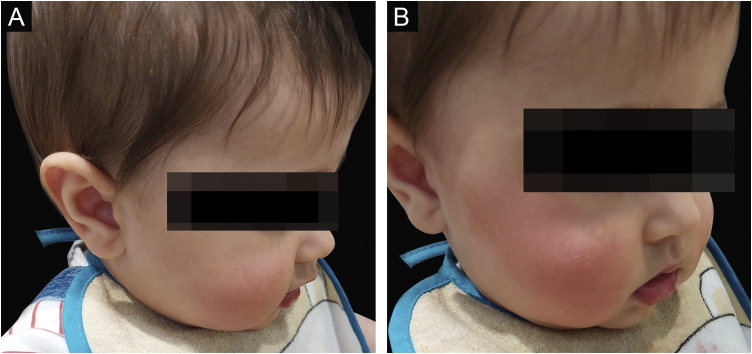
Before (A) and after (B) the provocation test in the clinic.

## What is your diagnosis?

a Frey syndrome,
b Fifth disease,
c Oral allergy syndrome,
d Mast cell activation syndrome.

## Discussion

The clinical history and provocation test are compatible with Frey syndrome.

Frey syndrome is a parasympathetic auriculotemporal nerve dysfunction, characterized by a transient flushing, localized warmth, and sometimes sweating of the face in the territory of the auriculotemporal nerve, a branch of the mandibular nerve of the trigeminal nerve complex. Symptoms classically occur in response to olfactory or gustatory stimuli, particularly with acidic, spicy, and sour foods.^1^, ^2^

In adults, it is a frequent complication of parotid surgery, but in children, it is very rare. As such, especially in bilateral cases, it is often misdiagnosed as a food allergy, prompting unnecessary diagnostic testing and treatment. A recent review showed that the diagnosis was made in only 20% of children at the first consultation and inappropriate dietary restriction was prescribed in 21%.^3^

In children, the majority (around 70%) of reported cases are unilateral, while associated sweating is rare (present in about 10%, in contrast to the adult forms, in which it is often present).^3^ The pathophysiology is incompletely understood. The prevailing hypothesis in unilateral forms is a traumatic lesion of the nerve fibers, followed by aberrant regeneration of the parasympathetic fibers into the skin, perhaps via sympathetic gains. This trauma could be neonatal, in instrumented delivery with forceps or ventouse, or postnatal.^4^ Bilateral forms appear to be largely idiopathic, but a familial bilateral syndrome has already been described.^5^

The outcome is overall favorable in unilateral cases, with regression in around 57% of cases, at a median age of 27 months.^3^ No further workup or treatment is recommended.

The authors report a classical Frey syndrome in childhood, after a forceps-assisted delivery, and with the absence of hyperhidrosis. The mother was reassured, and no specific treatment was provided. In contrast to the adult postsurgical form, Frey syndrome in children is rare and often constitutes a diagnostic challenge. In this case, the authors aim to increase familiarity with this benign condition, to avoid misdiagnosis and unnecessary procedures.

## Financial support

None declared.

## Authors' contributions

Ana Gusmão Palmeiro: Conceptualization; Methodology; Investigation; Data curation; Resources; Original draft preparation; Supervision.

Laura Azurara: Validation; Formal analysis; Review and editing.

Bernardo Pimentel: Validation; Formal analysis; Review and editing.

Cristina Amaro: Conceptualization; Validation; Formal analysis; Review and editing.

## Conflicts of interest

None declared.
